# Cytogenetic Abnormalities and Their Impact on Acute Myeloid Leukemia Outcomes Following Allogeneic Hematopoietic Stem Cell Transplantation: A Single‐Center Retrospective Study

**DOI:** 10.1002/hsr2.70914

**Published:** 2025-06-20

**Authors:** Amir Abbas Rashidi, Amir Kasaeian, Kamran Alimoghadam, Maryam Noori, Hediyeh Alemi, Ghazal Seghatoleslami, Oveis Salehi, Ardeshir Ghavamzadeh, Mohammad Vaezi, Seyed Asadollah Mousavi, Hossein Kamranzadeh, Davood Babakhani, Soroush Rad, Maryam Barkhordar, Sahar Tavakoli Shiraji, Marjan Yaghmaie

**Affiliations:** ^1^ Hematology, Oncology and Stem Cell Transplantation Research Center, Research Institute for Oncology, Hematology and Cell Therapy, Shariati Hospital Tehran University of Medical Sciences Tehran Iran; ^2^ Liver and Pancreatobiliary Diseases Research Center, Digestive Diseases Research Institute Tehran University of Medical Sciences Tehran Iran; ^3^ Digestive Oncology Research Center, Digestive Diseases Research Institute Tehran University of Medical Sciences Tehran Iran; ^4^ Research Center for Chronic Inflammatory Diseases Tehran University of Medical Sciences Tehran Iran; ^5^ Cell Therapy and Hematopoietic Stem Cell Transplantation Research Center, Research Institute for Oncology, Hematology, and Cell Therapy Tehran University of Medical Sciences Tehran Iran; ^6^ Student Research Committee, School of Medicine Iran University of Medical Sciences Tehran Iran; ^7^ Student Research Committee, Faculty of Medicine Mashhad University of Medical Sciences Mashhad Iran; ^8^ ATMP Department, Breast Cancer Research Center Motamed Cancer Institute, ACECR Tehran Iran; ^9^ Hematologic Malignancies Research Center Research Institute for Oncology, Hematology and Cell Therapy Tehran University of Medical Sciences Tehran Iran

**Keywords:** acute myeloid leukemia, allogeneic hematopoietic stem cell transplantation, chromosome aberrations, cytogenetic abnormalities, prognostics

## Abstract

**Background and Aims:**

Cytogenetic abnormalities at diagnosis are detected in over half of adult patients with acute myeloid leukemia (AML). This study aimed to evaluate the impact of these abnormalities on the outcomes of AML patients undergoing allogeneic hematopoietic stem cell transplantation (HSCT) in an Iranian cohort.

**Methods:**

This retrospective study included 206 AML patients aged over 15 who underwent allogeneic HSCT at a single center. Cytogenetic abnormalities were identified from medical records, and survival outcomes, including overall survival (OS) and leukemia‐free survival (LFS), were assessed across different cytogenetic risk groups. Multivariable analyses were adjusted for age, sex, donor type, time from diagnosis to transplant, and disease status at transplant.

**Results:**

The median follow‐up duration was 46.75 months. 1‐, 3‐, and 5‐year OS rates were 80.0%, 65.9%, and 58.5%, respectively, with significant differences based on cytogenetic risk. Patients with −7, 7q abnormalities, +8, complex karyotype, and monosomal karyotype exhibited shorter OS, classifying them as adverse‐risk. Patients with normal karyotype, inversion (16), and t(16;16) were intermediate‐risk. LFS at 1, 3, and 5 years was 74.6%, 64.5%, and 54.1%, respectively, with adverse cytogenetic profiles significantly associated with shorter LFS.

**Conclusions:**

This study demonstrates the prognostic significance of cytogenetic profiles in Iranian AML patients post‐HSCT. Results underscore the importance of cytogenetic stratification in guiding treatment decisions, suggesting that risk‐adapted guidelines may benefit specific populations. Future prospective studies on survival outcomes in Iranian AML patients could refine evidence‐based treatment guidelines.

## 1 Introduction

Acute myeloid leukemia (AML) is a heterogeneous neoplastic disorder characterized by various clinical characteristics and chromosomal alterations in the clonal undifferentiated hematopoietic cells [1]. Despite its aggressive nature and typically poor prognosis, several factors have been identified as predictive of survival in AML patients [2]. Among these factors, cytogenetic abnormalities are particularly prominent, detected in 50%–60% of newly diagnosed adult AML patients [3]. These pretreatment cytogenetic abnormalities have emerged as a paramount prognostic factor for AML, and consequently, they are instrumental in shaping contemporary risk‐stratified treatment guidelines [4]. Furthermore, our advancing knowledge about chromosomal aberrations in AML has provided a more profound insight into the disease's pathophysiology and progression.

Cytogenetic abnormalities assessed at early diagnosis are essential in clinical practice, helping predict outcomes and influence treatment strategies, especially for post‐remission therapies in AML. Research by various global collaborative groups has stratified AML patients into favorable, intermediate, and adverse risk based on their cytogenetic abnormalities [5, 6, 7, 8]. Over the past two decades, the increased use of hematopoietic stem cell transplantation (HSCT) has emerged as a pivotal factor in enhancing the prognosis of AML patients [9, 10]. Given HSCT's immunologic foundation, cytogenetic abnormalities may affect prognostic outcomes differently than in conventional chemotherapy [11]. Notably, the majority of patients examined in the aforementioned studies did not receive HSCT either post‐remission or as salvage therapy [7, 8]. Subsequent studies were specifically designed to assess the prognostic implications of cytogenetic changes in AML patients who underwent HSCT [12, 13, 14].

Some studies have correlated cytogenetic findings with post‐HSCT survival outcomes [14, 15, 16, 17], while others found no significant association [18, 19]. Additionally, the influence of cytogenetic abnormalities on relapse risk and incidence after HSCT is not yet fully understood. The present study, representing the largest analysis of cytogenetic status in AML patients undergoing allogeneic HSCT in Iran, aims to evaluate the prognostic impact of pretreatment cytogenetic abnormalities on post‐HSCT outcomes.

## 2 Patients and Methods

### 2.1 Patients

The medical records of all patients with AML who underwent HSCT at Shariati Hospital in Tehran, Iran, between March 2011 and May 2015 were reviewed. Patients were included in this retrospective study if they were 15 years or older at the time of transplantation, underwent their first allogeneic HSCT, and had their cytogenetic status at the time of diagnosis fully documented in their medical records. Bone marrow cytogenetic analysis was not repeated at the time of HSCT, and minimal residual disease (MRD) evaluation was unavailable for this cohort. Patients with acute promyelocytic leukemia or prior myelodysplastic syndromes, as well as those who had previously received autologous or allogeneic HSCT, were excluded. It should be noted that before 2016, all patients with a fully matched sibling donor at our center underwent allogeneic HSCT, regardless of cytogenetic analysis.

This study was conducted in accordance with the Declaration of Helsinki and received ethical approval from the Institutional Review Board (IRB) of the Hematology, Oncology, and Stem Cell Transplantation Research Center at Tehran University of Medical Sciences (Approval Reference: IR.TUMS.REC.1395.2830). Informed consent was obtained from all participants before their enrollment, with each participant providing consent for their clinical data to be used for research purposes. For patients under 18, informed consent was obtained from a parent or legal guardian.

### 2.2 Cytogenetics

At the time of diagnosis, standard cytogenetic analysis was performed on metaphase cells from bone marrow aspirate samples using standard methods. According to the International System for Human Cytogenetic Nomenclature [20], a cytogenetic abnormality was deemed clonal if at least two metaphases exhibited the same aberration, whether as a structural abnormality or an additional chromosome. A clonal monosomy was defined by the presence of monosomy in at least three metaphases. A minimum of 20 metaphases from the bone marrow was required to classify a karyotype as normal. An analysis of fewer than 20 normal metaphases was considered a failure. A complex karyotype was defined as the presence of three or more unrelated cytogenetic abnormalities [7], and a monosomal karyotype (MK) was identified when at least two autosomal monosomies or a single autosomal monosomy combined with at least one structural abnormality were present [21].

### 2.3 Hematopoietic Stem Cell Transplantation

A myeloablative regimen, consisting of intravenous Busulfan, 4 mg/kg/day from Day‐6 to Day‐3 and Cyclophosphamide, 60 mg/kg/day on Day‐2 and Day‐1, was used for conditioning before HSCT. Only two patients received a reduced‐intensity conditioning regimen. The stem cell source for all patients was peripheral blood. Recipients of grafts from matched unrelated donors, other related donors, and haploidentical donors were administered anti‐thymocyte globulin before transplantation. Cyclosporine and Methotrexate were administered for graft‐versus‐host disease (GVHD) prophylaxis.

### 2.4 Statistical Analysis

Overall survival (OS) was defined as the time from HSCT to death, with patients censored at the date of their last contact if alive. Leukemia‐free survival (LFS) was defined as survival without evidence of relapse or progression. The cumulative incidence of relapse (CIR) was considered any event related to the recurrence of leukemia. The probability of OS and LFS were estimated using the Kaplan–Meier method and compared using the log‐rank test. The Cox proportional hazards regression model was employed for multivariate analysis, and hazard ratios (HRs) were calculated with their corresponding 95% confidence intervals (CIs). Patients were categorized based on OS into three risk groups: favorable, intermediate, and adverse. Individuals with a normal karyotype or comparable OS durations were classified as intermediate risk. Those with cytogenetic abnormalities, whose OS was significantly shorter compared to the normal group, were categorized as adverse risk. Conversely, patients with cytogenetic abnormalities and notably better OS were designated as the favorable risk group. Furthermore, we evaluated whether the occurrence of secondary abnormalities or a complex karyotype, featuring four or more abnormalities, affected the prognosis of patients with core‐binding factor AML (CBF‐AML). CBF‐AML is characterized by the existence of t(8;21) or inversion (16)/t(16;16). A *p* value less than 0.05 was considered statistically significant.

While multiple univariate analyses were initially conducted to assess associations, we addressed the potential for multiple testing concerns by performing multivariable analyses for each cytogenetic abnormality group. These multivariable models adjusted for relevant confounding factors, strengthening the validity of our findings and reducing the risk of Type I errors. We performed all statistical analyses using STATA version 13.

## 3 Results

### 3.1 Patient Characteristics

The medical data of 369 consecutive AML patients who underwent allogeneic HSCT were reviewed. Of these, 163 were deemed ineligible and excluded due to the unavailability of pretreatment cytogenetic status (*n* = 113), age under 15 (*n* = 26), undergoing autologous HSCT (*n* = 20), or having more than one transplantation (*n *= 4). Among the 206 enrolled patients, 124 (60.2%) had a normal karyotype, and 82 (39.8%) exhibited one or more cytogenetic abnormalities. The baseline characteristics of patients are presented in Table 1. The median age at transplantation was 33 years (range: 15–64) with 128 (62.1%) male participation. AML M2 was the most common French‐American‐British (FAB) classification subtype, diagnosed in 73 (35.4%) patients. Myeloablative conditioning regimens were utilized in 204 (99%) patients, while reduced‐intensity regimens were used in 2 (1%) patients. Matched donors (sibling/relative/unrelated) were available for 181 (77.9%) patients, whereas 25 (12.1%) had mismatched donors. The median interval between diagnosis and HSCT was 7.4 months (range: 1.2–49.7 months), and the majority of patients (74.7%) underwent HSCT in the first complete remission (CR1).

**Table 1:** General and clinical profile of the AML patients.

Characteristics of the patients (*n* = 206)		Number (%)
Age, years	15–60	203 (98.5)
	> 60	3 (1.5)
Sex	Male	128 (62.1)
	Female	78 (37.9)
FAB subtype	M0	2 (0.9)
	M1	22 (10.6)
	M1/2	1 (0.4)
	M2	73 (35.4)
	M4	48 (23.3)
	M5	23 (11.1)
	M6	2 (0.9)
	M7	1 (0.4)
	Unclassified	34 (17)
Cytogenetic	Normal	124 (60.2)
	Abnormal	82 (39.8)
Conditioning regimen	Myeloablative	204 (99)
	Reduced intensity regimen	2 (1)
Donor type	Match (sibling/relative/unrelated)	181 (77.9)
	Miss‐match (sibling/unrelated)	25 (12.1)
Disease status at HSCT	CR1	154 (74.7)
	CR2	41 (20)
	Blast > 5%	11 (5.3)
Time from diagnosis to HSCT, months	< 6	80 (38.8)
	≥ 6	126 (61.2)

Abbreviations: CR, complete remission; HSCT, hematopoietic stem cell transplantation.

### 3.2 Survival Analysis

#### 3.2.1 Overall Survival

**Figure 1:**
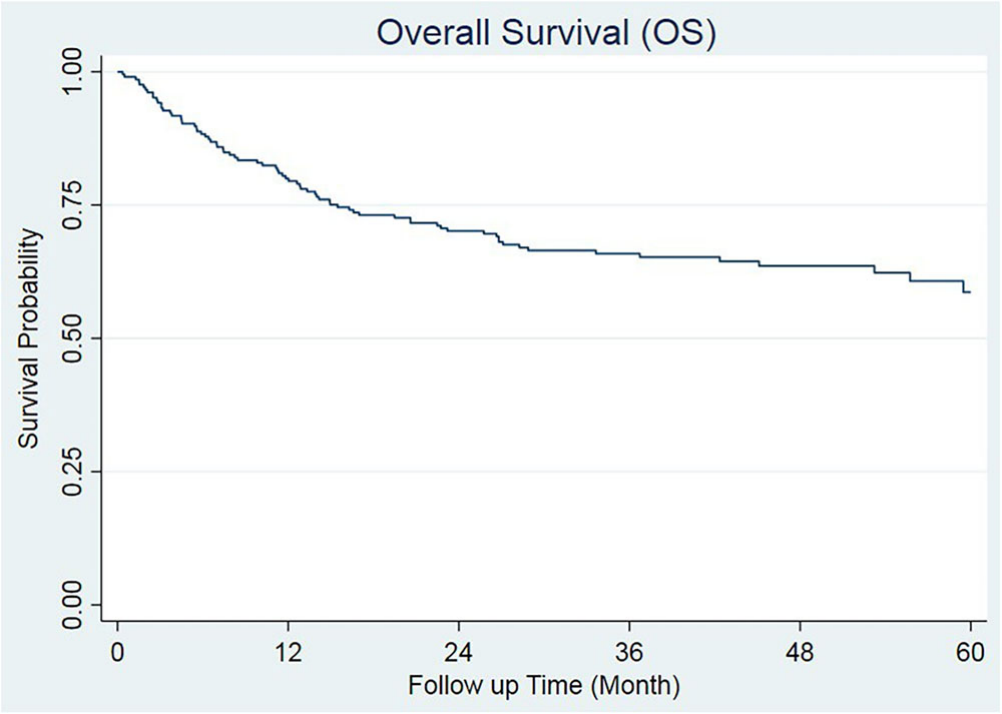
Kaplan–Meier Survival curve: overall survival in AML patients following HSCT.

The median follow‐up duration was 46.75 months (95% CI: 44.09–50.36). The 1‐, 3‐, and 5‐year OS rates for all patients were 80.0% (95% CI: 73.8–84.8), 65.9% (95% CI: 58.8–72.0), and 58.5% (95% CI: 49.6–66.4), respectively (Figure 1). The OS probability for each cytogenetic group is summarized in Table 2.

**Table 2:** Probability of overall survival stratified by cytogenetic abnormalities.

Groups	Number	1 year‐OS (95% CI)	3 year‐OS (95% CI)	5 year‐OS (95% CI)	*p* value
Total	206	80.0 (73.8–84.8)	65.9 (58.9–72.0)	58.5 (49.6–66.4)	—
Normal cytogenetics	124	83.8 (76.0–89.2)	74.6 (65.9–81.4)	70.0 (60.0–78.0)	—
t (8;21)	9	76.2 (33.2–93.5)	50.8 (15.7–78.1)	30.5 (3.6–63.3)	0.06
Inversion (16) and t (16;16)	8	75.0 (31.5–93.1)	62.5 (22.9–86.1)	NA	0.54
13 abnormality	7	100	57.1 (17.2–83.7)	0	0.17
−20 and 20q abnormality	7	71.4 (25.8–92.0)	57.1 (17.2–83.2)	NA	0.08
+21	6	83.3 (27.3–97.5)	83.3 (27.3–97.5)	NA	0.61
−Y	5	80.0 (23.4–96.9)	80.0 (23.4–96.9)	80.0 (23.4–96.9)	0.76
−7, 7q abnormality	10	60.0 (25.3–82.7)	30.0 (0.7–57.8)	30.0 (0.7–57.8)	0.001
+8	9	66.7 (28.2–87.8)	44.4 (13.6–71.9)	22.2 (1.4–58.8)	0.008
3q abnormality, inversion (3), t (3;3)	7	57.1 (17.2–83.7)	14.3 (0.7–46.5)	14.3 (0.7–46.5)	< 0.001
t (6;9)	5	40.0 (5.2–58.2)	20.0 (0.8–58.2)	20.0 (0.8–58.2)	0.005
1p and 1q abnormality	5	60.0 (12.6–88.2)	40.0 (21.9–75.3)	NA	0.04
Del and add 9q	4	66.7 (19.5–90.4)	16.7 (0.8–51.7)	NA	< 0.001
t (11q23) excluding (11;19) and (9;11)	4	25.0 (0.9–66.5)	25.0 (0.9–66.5)	NA	0.007
17p abnormality	4	75.0 (12.8–96.0)	50.0 (5.8–84.5)	0	0.03
Del and add 5q	3	33.3 (0.9–77.4)	0	NA	< 0.001
Complex karyotype	17	64.7 (37.7–82.3)	33.6 (12.9–56.0)	0	< 0.001
MK+	8	62.5 (22.9–86.1)	50.0 (15.2–77.5)	25.0 (1.4–63.6)	0.03
Other abnormalities	16	81.2 (52.5–93.5)	75.0 (46.3–89.9)	60.0 (24.2–83.2)	0.82

Abbreviations: Add, addition; CI, confidence interval; Del, deletion; MK, monosomal karyotype; NA, not available; OS, overall survival.

The OS analysis showed that, in comparison to the normal karyotype group, none of the other groups exhibited significantly better survival. Patients with specific cytogenetic abnormalities such as −7, 7q abnormality, +8, 3q abnormality, inversion (3), t(3;3), t(6;9), deletion and addition of 9q, t(11q23) excluding t(9;11), 17p abnormality, deletion and addition 5q, complex karyotype, and monosomal karyotype had significantly shorter survival and were classified as patients with adverse‐risk cytogenetics. Conversely, the probability of survival in patients with t(8;21), inversion (16) and t(16;16), 13q abnormality, −20 and 20q abnormality, +21, −Y, 1p and 1q abnormalities was not significantly different from that of the normal karyotype group and, therefore, they were classified as patients with intermediate‐risk cytogenetics. Multivariable analysis, adjusted for age, sex, donor matching, time interval between diagnosis and transplant, and disease status at transplant, only changed the classification of t(8;21) to adverse‐risk cytogenetics (Table 3).

**Table 3:** Univariable and multivariable analysis of overall survival based on the cytogenetic abnormalities.

Groups	Number	Unadjusted HR (95% CI)	*p* value	Adjusted HR* (95% CI)	*p* value
t (8;21)	9	2.38 (0.92–6.11)	0.071	2.63 (1.01–6.78)	0.04
Inversion (16) and t (16;16)	8	1.43 (0.44–4.67)	0.549	1.47 (0.45–4.81)	0.51
13 abnormality	7	2.00 (0.71–5.66)	0.187	2.05 (0.72–5.81)	0.17
−20 and 20q abnormality	7	2.44 (0.86–6.89)	0.092	2.29 (0.81–6.48)	0.11
+21	6	0.60 (0.08–4.39)	0.617	0.54 (0.07–4.01)	0.55
−Y	5	0.73 (0.10–5.36)	0.761	0.65 (0.08–4.80)	0.67
1p and 1q abnormality	5	3.08 (0.94–10.10)	0.062	3.24 (0.98–10.68)	0.05
−7, 7q abnormality	10	3.52 (1.54–8.01)	0.003	3.60 (1.57–8.24)	0.002
+8	9	3.02 (1.26–7.22)	0.013	3.28 (1.37–7.88)	0.008
3q abnormality, inversion (3), t (3;3)	7	5.13 (2.12–12.39)	< 0.001	4.78 (1.97–11.55)	0.001
t (6;9)	5	3.89 (1.37–11.03)	0.010	3.48 (1.16–10.43)	0.02
Del and add 9q	4	5.08 (2.40–14.00)	< 0.001	4.99 (1.69–14.74)	0.004
t (11q23) excluding (11;19) and (9;11)	4	4.44 (1.34–14.69)	0.014	4.83 (1.45–16.06)	0.01
17p abnormality	4	3.29 (1.00–10.75)	0.048	4.00 (1.20–13.28)	0.02
Del and add 5q	3	7.75 (2.30–26.13)	0.001	6.74 (1.99–22.87)	0.002
Complex karyotype	17	3.36 (1.73–6.52)	< 0.001	3.17 (1.63–6.18)	0.001
MK+	8	2.71 (1.06–6.97)	0.037	3.02 (1.16–7.82)	0.02
Other abnormalities	16	1.10 (0.43–2.83)	0.829	0.97 (0.37–2.53)	0.96

Abbreviations: Add, addition; CI, confidence interval; Del, deletion; HR, hazard ratio; MK, monosomal karyotype; NA, not available.

* HR adjusted for age, sex, donor matching, time interval between diagnosis and transplant, and disease status at transplant.

#### 3.2.2 Leukemia‐Free Survival

**Figure 2:**
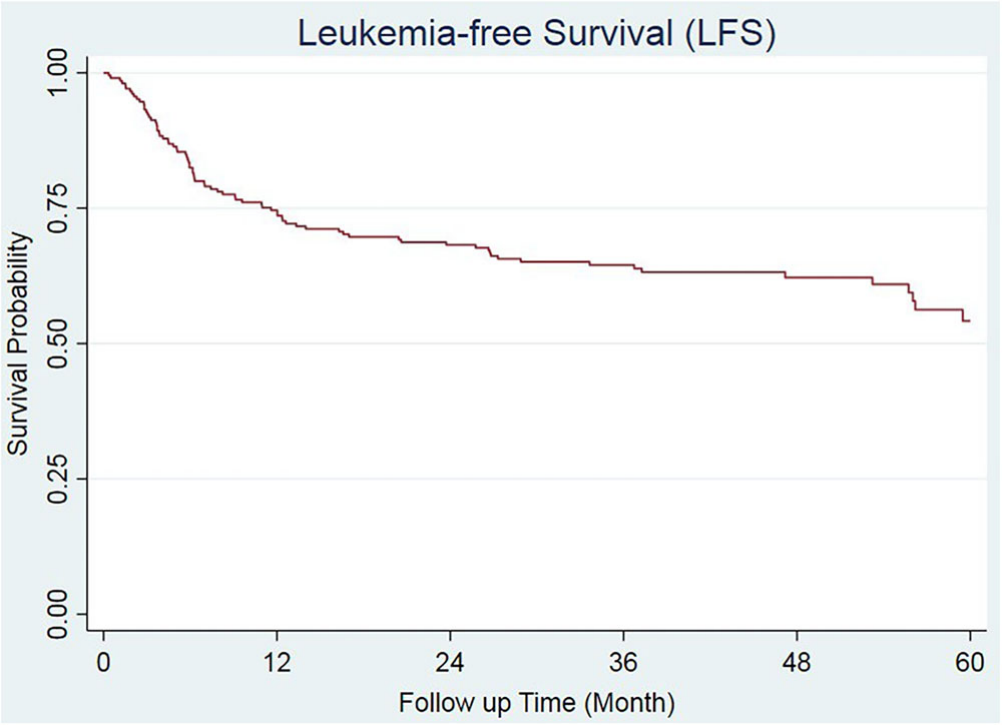
Kaplan–Meier survival curve: Leukemia‐free survival in AML patients following HSCT.

In the context of LFS analysis, the first year's LFS stood at 74.6% (95% CI 68.0–80.0) for AML patients following allogenic transplantation. Furthermore, the 3‐ and 5‐year LFS were estimated to be 64.5% (57.5–70.7) and 54.1% (44.6–62.6), respectively (Figure 2). The likelihood of LFS for all cytogenetic groups is presented in Table 4.

**Table 4:** Probability of leukemia‐free survival stratified by cytogenetic abnormalities.

Groups	Number	1 year‐LFS (95% CI)	3 year‐LFS (95% CI)	5 year‐LFS (95% CI)	*p*‐value
Total	206	74.6 (68.0–80.0)	64.5 (57.5–70.7)	54.1 (44.6–62.6)	‐‐‐
Normal cytogenetics	124	81.4 (73.3–87.2)	72.3 (63.4–79.3)	67.4 (57.3–75.7)	‐‐‐
t (8;21)	9	77.8 (36.5–93.9)	51.8 (16.4–78.8)	31.1 (3.7–66.1)	0.09
Inversion (16) and t (16;16)	8	62.5 (22.9–86.1)	62.5 (22.9–86.1)	NA	0.61
13 abnormality	7	85.7 (33.4–97.9)	57.1 (17.2–83.7)	0.00	0.23
−20 and 20q abnormality	7	57.1 (17.2–83.7)	57.1 (17.2–83.7)	NA	0.11
+21	6	83.3 (27.3–97.5)	83.3 (27.3–97.5)	NA	0.56
−Y	5	80.0 (20.4–96.9)	80.0 (20.4–96.9)	80.0 (20.4–96.9)	0.69
−7, 7q abnormality	10	30.0 (7.1–57.8)	30.0 (7.1–57.8)	30.0 (7.1–57.8)	0.001
+8	9	55.6 (20.4–80.4)	44.4 (13.6–71.9)	NA	0.001
3q abnormality, inversion (3), t (3;3)	7	28.6 (4.1–61.1)	14.3 (0.7–46.5)	14.3 (0.7–46.5)	< 0.001
t (6;9)	5	20.0 (0.8–58.2)	20.0 (0.8–58.2)	20.0 (0.8–58.2)	0.01
1p and 1q abnormality	5	40.0 (5.2–75.3)	40.0 (5.2–75.3)	NA	0.05
Del and add 9q	4	33.3 (4.6–67.6)	16.7 (0.8–51.7)	NA	< 0.001
t (11q23) excluding (11;19) and (9;11)	4	25.0 (0.9–66.5)	25.0 (0.9–66.5)	NA	0.01
17p abnormality	4	50.0 (5.8–84.5)	50.0 (5.8–84.5)	0	0.03
Del and add 5q	3	0.00*	NA	NA	< 0.001
Complex karyotype	17	47.1 (23.0–68.0)	34.0 (13.2–56.3)	0	< 0.001
MK+	8	50.0 (15.2–77.5)	50.0 (15.2–77.5)	25.0 (1.4–63.6)	0.04
Other abnormalities	16	81.2 (52.5–93.5)	75.0 (46.3–89.8)	45.0 (12.3–73.7)	0.71

Abbreviations: Add, addition; CI, confidence interval; Del, deletion; LFS, leukemia free survival; MK, monosomal karyotype; NA, not available;

* 11‐month survival, did not reach the first year of follow‐up.

Consistent with OS outcomes in the multivariable analysis, patients with −7, 7q abnormality, +8, 3q abnormalities, inversion (3), t(3;3), t(6;9), deletion and addition of 9q, t(11q23) (excluding t(9;11)), 17p abnormalities, deletion and addition of 5q, complex karyotype, and monosomal karyotype exhibited shorter LFS than the normal group. Meanwhile, patients with t(8;21), inversion (16) and t(16;16), 13q abnormality, −20 and 20q abnormalities, +21, −Y, and 1p and 1q abnormalities displayed outcomes comparable to patients with normal cytogenetic profiles in multivariable analysis (Table 5).

**Table 5:** Univariable and multivariable analysis of leukemia‐free survival based on the cytogenetic abnormalities.

Groups	Number	Unadjusted HR (95% CI)	*p* value	Adjusted HR* (95% CI)	*p* value
t (8;21)	9	2.16 (0.84–5.52)	0.106	2.36 (0.92–6.08)	0.07
Inversion (16) and t (16;16)	8	1.34 (0.41–4.37)	0.619	1.34 (0.41–4.37)	0.61
13 abnormality	7	1.85 (0.66–5.21)	0.24	1.90 (0.67–5.34)	0.22
−20 and 20q abnormality	7	2.23 (0.79–6.27)	0.128	2.14 (0.76–6.04)	0.14
+21	6	0.56 (0.07–4.09)	0.569	0.52 (0.07–3.85)	0.52
−Y	5	0.67 (0.09–4.91)	0.697	0.61 (0.08–4.52)	0.63
1p and 1q abnormality	5	3.00 (0.92–9.78)	0.068	3.25 (0.99–10.67)	0.052
−7, 7q abnormality	10	3.50 (1.54–7.93)	0.003	3.58 (1.57–8.14)	0.002
+8	9	3.34 (1.48–7.52)	0.003	3.34 (1.48–7.52)	0.003
3q abnormality, inversion (3), t (3;3)	7	5.15 (2.14–12.38)	< 0.001	4.93 (2.05–11.87)	< 0.001
t (6;9)	5	3.36 (1.19–9.46)	0.022	3.02 (1.03–8.89)	0.04
Del and add 9q	4	6.47 (2.69–15.52)	< 0.001	5.09 (1.85–13.99)	0.002
11q23 and 11q abnormality	4	3.87 (1.18–12.71)	0.025	4.12 (1.25–13.58)	0.02
17p abnormality	4	3.24 (0.99–10.55)	0.05	3.90 (1.17–12.95)	0.02
Del and add 5q	3	8.10 (2.41–27.16)	0.001	7.33 (2.17–24.75)	0.001
4+ abnormalities	17	3.22 (1.67–6.22)	< 0.001	3.13 (1.62–6.05)	0.001
MK+	8	2.47 (0.96–6.30)	0.058	2.71 (1.05–6.99)	0.03
Other abnormalities [16]	16	1.18 (0.49–2.80)	0.702	1.06 (0.44–2.56)	0.88

Abbreviations: Add, addition; CI, confidence interval; Del, deletion; HR, hazard ratio; MK, monosomal karyotype; NA, not available.

* HR adjusted for age, sex, donor matching, time interval between diagnosis and transplant, and disease status at transplant.

#### 3.2.3 Cumulative Incidence of Relapse

The cumulative AML relapse incidences at 1, 3 and 5 years following allogenic transplantation were 16.1% (95% CI 11.5–21.5), 20.1% (95% CI 14.9–25.9), and 24.95% (18.2–32.3), respectively. The CIR for all cytogenetic groups is presented in Table 6.

**Table 6:** Relapse Incidence stratified by cytogenetic abnormalities.

Groups	Number	1 year‐OS (95% CI)	3 year‐OS (95% CI)	5 year‐OS (95% CI)	*p* value
Total	206	16.12 (11.45–21.50)	20.12 (14.91– 25.89)	24.95 (18.17–32.31)	‐‐‐
Normal cytogenetics	124	10.55 (5.90–16.73)	13.87 (8.43– 20.63)	15.52 (9.43–22.99)	0.002
t (8;21)	9	11.11 (0.45–40.89)	37.04 (6.73–69.38)	37.04 (6.73–69.38)	0.11
Inversion (16) and t (16;16)	8	12.50 (0.46–44.82)	12.50 (0.46–44.82)	NA	0.85
13 abnormality	7	14.29 (0.49–49.13)	28.57 (3.12–63.60)	NA	0.36
−20 and 20q abnormality	7	42.86 (7.58–75.70)	42.86 (7.58–75.70)	NA	0.04
+21	6	16.67 (0.47–54.88)	16.67 (0.47–54.88)	NA	0.81
−Y	5	0.00	0.00	0.00	0.36
−7, 7q abnormality	10	60.00 (21.79–84.26)	60.00 (21.79–84.26)	60.00 (21.79–84.26)	< 0.001
+8	9	33.33 (6.68–64.01)	44.44 (11.50–73.78)	NA	< 0.001
3q abnormality, inversion (3), t (3;3)	7	57.14 (12.11–86.21)	57.14 (12.11–86.21)	57.14 (12.11–86.21)	0.003
t (6;9)	5	20.00 (0.43–62.11)	20.00 (0.43–62.11)	20.00 (0.43–62.11)	0.74
1p and 1q abnormality	5	60.00 (6.72–90.79)	60.00 (6.72–90.79)	NA	0.003
Del and add 9q	4	50.00 (6.88–83.57)	50.00 (6.88–83.57)	NA	< 0.001
t (11q23) excluding (11;19) and (9;11)	4	75.00 (1.71–97.99)	NA	NA	0.001
17p abnormality	4	50.00 (2.26–88.10)	50.00 (2.26–88.10)	NA	0.04
Del and add 5q	3	NA	NA	NA	< 0.001
Complex karyotype	17	35.29 (13.72–57.92)	42.02 (17.75–64.73)	NA	0.005
MK+	8	37.50 (6.92–69.75)	37.50 (6.92–69.75)	37.50 (6.92–69.75)	0.07
Other abnormalities	16	6.25 (0.36–25.48)	12.50 (1.89–33.64)	27.50 (3.62–60.52)	0.72

Abbreviations: Add, addition; CI, confidence interval; HR, hazard ratio; Del, deletion; MK: monosomal karyotype; NA, not available, since those patients with this cytogenetic abnormalities have no more follow‐up.

The multivariable analysis of CIR demonstrated that patients with t(8;21), 13q abnormalities, −20 and 20q abnormalities, 1p and 1q abnormalities, −7, 7q abnormalities, +8, 3q abnormalities, inversion (3), t(3;3), deletion and addition 9q, t(11q23) excluding t(9;11), 17p abnormalities, deletion and addition 5q, complex karyotype, and monosomal karyotype exhibited higher relapse incidence compared to the normal group. Patients with inversion (16) and t(16;16), +21, and t(6;9) showed no difference in relapse incidence relative to patients with a normal karyotype group (Table 7).

**Table 7:** Univariable and multivariable analysis of relapse incidence based on the cytogenetic abnormalities.

Groups	Number	Unadjusted HR (95% CI)	*p* value	Adjusted HR* (95% CI)	*p* value
t (8;21)	9	2.66 (1.16–6.09)	0.021	5.04 (1.74–14.58)	0.003
Inversion (16) and t (16;16)	8	0.83 (0.2–3.46)	0.807	0.97 (0.21–4.49)	0.97
13 abnormality	7	1.98 (0.73–5.31)	0.174	2.83 (1.01–7.9)	0.04
−20 and 20q abnormality	7	3.62 (1.54–8.51)	0.003	6.03 (2.74–13.25)	< 0.001
+21	6	1.27 (0.28–5.75)	0.751	2.05 (0.43–9.72)	0.36
−Y	5	NA	NA	NA	‐‐‐
1p and 1q abnormality	5	6.63 (2.57–17.14)	< 0.001	17.93 (5.91–54.35)	< 0.001
−7, 7q abnormality	10	5.98 (3.11–11.52)	< 0.001	7.32 (3.89–13.80)	< 0.001
+8	9	6.07 (3.39–10.89)	< 0.001	7.18 (4.13–12.5)	< 0.001
3q abnormality, inversion (3), t (3;3)	7	5.42 (2.50–11.76)	< 0.001	6.34 (2.82–14.21)	< 0.001
t (6;9)	5	1.47 (0.33–6.55)	0.612	1.29 (0.45–3.72)	0.62
Del and add 9q	4	6.61 (3.15–13.86)	< 0.001	3.32 (1.10–9.98)	0.03
t (11q23) excluding (11;19) and (9;11)	4	8.24 (3.74–18.13)	< 0.001	6.01 (2.38–15.16)	< 0.001
17p abnormality	4	4.85 (1.59–14.77)	0.005	18.81 (5.38–65.80)	< 0.001
Del and add 5q	3	16.02 (8.56–29.99)	< 0.001	16.58 (6.81–40.35)	< 0.001
Complex karyotype	17	3.43 (1.86–6.34)	< 0.001	6.67 (3.59–12.40)	< 0.001
MK+	8	3.03 (1.26–7.24)	0.013	8.72 (4.12–18.46)	< 0.001
Other abnormalities	16	1.24 (0.54–2.87)	0.603	1.65 (0.70–3.90)	0.24

Abbreviations: Add, addition; CI, confidence interval; Del, deletion; HR, hazard ratio; MK, monosomal karyotype; NA, not available, since there was no relapse in this group of cytogenetic abnormality, the HR was not estimable.

* HR adjusted for age, sex, donor matching, time interval between diagnosis and transplant, and disease status at transplant.

#### 3.2.4 Significance of Additional Cytogenetic Aberrations in Core‐Binding Factor AML

This study also assessed whether secondary karyotype abnormalities or complex karyotypes with three or more abnormalities influenced outcomes in patients with CBF‐AML. Six patients had an isolated t(8;21) and seven had an inversion (16)/t(16;16). Patients with an isolated t(8;21) presented with OS comparable to those with a normal karyotype (adjusted HR 2.70, 95% CI 0.81–8.99). An improvement in OS was observed among patients with isolated inversion (16)/t(16;16) when compared to those with a normal karyotype, although the difference did not reach statistical significance (adjusted HR 0.88, 95% CI 0.19–3.94) (Supporting Information S1: Tables S1–S2). These findings indicate that the presence of secondary abnormalities or a complex karyotype with three or more abnormalities adversely affects the OS of patients with t(8;21) but do not significantly impact the OS of patients with inversion (16)/t(16;16).

In LFS analysis, patients with isolated t(8;21) or inversion (16)/t(16;16) continued to show nonsignificant changes in LFS relative to normal cytogenetic AML patients (adjusted HR 2.36, 95% CI 0.71–7.79 and adjusted HR 1.00, 95% CI 0.24–4.16, respectively). These results emphasize that neither secondary abnormalities nor a complex karyotype with four or more abnormalities has a negative impact on the LFS of patients with t(8;21) or inversion (16)/t(16;16) (Supporting Information S1: Tables S3–S4).

## 4 Discussion

This study is one of the first to analyze the outcomes for AML patients undergoing allogeneic HSCT based on cytogenetic subgroupings in Iran. Our findings provide strong evidence for the powerful prognostic impact of pretreatment cytogenetic abnormalities on the survival outcomes in AML patients post‐HSCT, even after adjusting for other prognostic factors. These results offer valuable insights for clinicians in their clinical practice, enabling the stratification of patients based on their cytogenetic profiles and the development of strategies to aid in selecting the most appropriate treatment.

In recent years, there has been a significant improvement in the outcomes of AML patients post‐HSCT. Studies have shown that recent transplant recipients demonstrate a 16% increase in LFS, accompanied by enhanced OS and reduced non‐relapsed mortality rates compared to earlier years [22]. Advancement in high‐resolution human leukocyte antigen (HLA)‐typing, improved GVHD prophylaxis, and the provision of enhanced supportive care have the potential to yield better outcomes for older patients or individuals with lower fitness levels [23]. However, despite these advancements, data on Iranian patients remain limited, emphasizing the need for reassessment of risk‐stratification guidelines tailored to Iranian AML patients.

Although our study does not include direct socioeconomic data, existing literature suggests that socioeconomic disparities play a significant role in AML outcomes. For instance, Berger et al. demonstrated that patients with lower socioeconomic status had worse survival outcomes, largely due to limited access to comprehensive care and advanced therapeutic options [24]. In regions with limited resources, socioeconomic factors may influence AML outcomes indirectly by affecting early diagnosis, supportive care, and access to post‐transplant care [25, 26, 27]. Collectively, these disparities in access to well‐equipped healthcare services for transplantation, insufficient HLA‐typing technologies, and inadequate post‐transplant supportive care may explain the reduced success of therapeutic approaches in AML patients with lower socioeconomic status.

Several studies verifying the impact of cytogenetics on the survival of AML patients have classified t(8;21), inversion (16), and t(16;16) as a favorable category [7, 14]. However, our study did not show improved survival for patients with these abnormalities when compared to those with a normal karyotype. This discrepancy may be attributed to the retrospective nature of our study, highlighting the potential for selection bias. It is important to note that the treatment choice for AML patients in the favorable risk groups does not typically involve HSCT, and these patients are generally not considered candidates for transplantation at CR1. For instance, among the eight patients with inversion (16) and t(16;16) in our study, three patients in CR2 and one in CR3 with blast exceeding 5% underwent HSCT, which may explain why these patients were not classified in the favorable risk group. Yanada and colleagues also noted the poor disease status of AML patients with t(8;21) or inversion (16)/t(16;16) at the time of transplantation as a rationale for observing only a slight difference in survival between AML patients in the favorable and intermediate risk groups [14]. Even after running a multivariate analysis adjusting for disease status, we found that the inversion (16) and t(16;16) karyotypes remained in the intermediate‐risk group, while the t(8;21) karyotype was reclassified as an adverse risk group. These findings suggest that additional biological factors, such as molecular mutations or secondary chromosomal changes, may further modulate prognosis in CBF‐AML. As Armand et al. also observed, having favorable cytogenetics may not reliably predict outcomes post‐HSCT [17]. Therefore, a more comprehensive approach to risk assessment is required, one that looks beyond standard classifications when making treatment decisions.

In line with our findings, several studies have reported that t(8;21) does not confer as favorable a prognosis as inversion [16] after HSCT and categorized it as intermediate cytogenetics [17, 28, 29, 30]. Nevertheless, it is worth noting that both t(8;21) and inversion [16] are still classified as a favorable risk group in the latest European Leukemia Network guideline updated in 2023 [31]. The discrepancy may be attributed to the absence of matching for FLT3 or NPM1 mutations in the control group, both of which have proven to possess prognostic significance in AML patients [31, 32]. Besides, although our control population comprised patients with no cytogenetic abnormality, they were heterogeneous in the context of molecular markers such as FLT3 and NPM1 mutations.

A growing body of evidence supports the notion that patients within each cytogenetic risk group, particularly those in the favorable and intermediate‐risk categories, exhibited varying outcomes when treated with chemotherapy [33, 34]. This variability may also extend to patients undergoing allogeneic HSCT. Notably, KIT mutations can occur in approximately one‐third of CBF‐AML patients [35]. These mutations are associated with an increased relapse rate and shorter OS, prompting clinicians to reclassify the risk group of these patients into the intermediate‐risk category [36, 37]. Additionally, it has been observed that the presence of internal tandem duplication of FLT3 gene (FLT3‐ITD) in patients with a normal karyotype undergoing allogeneic HSCT is correlated with a worse prognosis [38, 39]. Consequently, the 2017 European Leukemia Network recommended a risk classification system that integrates both cytogenetic and molecular profiles of AML patients, considering the mutational status of FLT3‐ITD, NPM1, CEBPA, and TP53 [40]. Considering the high proportion of normal cytogenetics (about 60%) observed in our study, one potential explanation could be the limited accessibility of high‐resolution karyotype analysis by G‐banding in many Iranian healthcare centers. This could lead to the underestimation of cytogenetic abnormalities. It is essential to note that the data for all of these gene mutations were not available for our patients; thus, these mutations were not included in our analysis. As a result, the prognostic significance and potential interactions between cytogenetic and molecular abnormalities should be addressed in future investigations.

In our analysis, patients with t(8;21) and additional chromosomal abnormalities exhibited lower OS compared to those with a normal karyotype. However, when we restricted the analysis to patients with isolated t(8;21), we observed comparable OS relative to patients with a normal karyotype. Conversely, the presence of additional abnormalities significantly impacted outcomes for patients with inversion (16) and t(16;16). In a related study, Han and colleagues aimed to identify specific aberrations that might influence the outcomes of nearly 500 CBF‐AML patients [41]. They found that 9q deletion and hyperdiploidy could extend the OS of AML patients with t(8;21); however, none of the other cytogenetic abnormalities had a significantly adverse impact on OS, which contrasts with our findings. Regarding the inversion [16] abnormality, additional trisomy 8 was associated with decreased OS, whereas other chromosomal abnormalities, except for trisomy 8, appeared to improve it [41]. In a German population study, 9q deletion or additional abnormalities did not substantially alter the outcomes of AML patients harboring t(8;21), while loss of the Y chromosome was associated with better OS [29]. In the context of inv(16)/t(16;16), any additional abnormalities significantly enhanced OS. These discrepancies suggest a need for larger studies to clarify the role of cytogenetic abnormalities in CBF‐AML patients.

Our study reports 1‐, 3‐, and 5‐year OS rates of 80.0%, 65.9%, and 58.5%, and 1‐, 3‐, and 5‐year LFS rates of 74.6%, 64.5%, and 54.1% in AML patients undergoing allogeneic HSCT. These findings are consistent with large‐scale registry data. For example, Mizuno et al. reported 5‐year OS rates of 66% for favorable‐risk, 61% for intermediate‐risk, and 47% for adverse‐risk AML patients post‐ HSCT [42]. Relapse remains the primary cause of treatment failure, which is directly reflected in both OS and LFS curves. The CIR in our cohort increased from 16.1% at 1 year to 20.1% at 3 years and 24.95% at 5 years, consistent with prior findings [43, 44]. The difference between OS and LFS observed in our study also reflects the enduring burden of non‐relapse mortality due to complications such as graft‐versus‐host disease, infections, or organ toxicity, which continues to be a challenge in the post‐transplant period.

We acknowledge that approximately 30% (116/365) of patients from the reviewed cohort were excluded due to missing pretreatment cytogenetic data. In our center, certain patients are referred solely for transplantation and may lack comprehensive diagnostic data, including cytogenetic analyses conducted before referral. This limitation reflects the referral pattern rather than data collection practices within our center. Despite this, the included cohort provides valuable insights into the prognostic significance of cytogenetic abnormalities in patients with available data, which is instrumental for understanding AML outcomes post‐transplant in this population. Several other limitations of this study must be considered when interpreting our findings. First, the retrospective nature of the study may have introduced selection bias because we exclusively included AML patients who had undergone HSCT. This potential bias might be particularly relevant among patients with AML harboring t(8;21), inversion (16), or t(16;16), as HCT is not typically performed in CR1 for these subgroups. Secondly, we did not ascertain the etiology of AML (i.e., therapy‐induced, post‐MDS, or de novo AML) at baseline, which is a well‐known prognostic factor among this patient population. Thirdly, we did not incorporate molecular prognostic factors in our analysis due to the unavailability of data for all patients. Fourthly, the limited number of patients for each cytogenetic abnormality diminishes the statistical power of the study, making it challenging to reliably detect differences. These findings need further validation in future studies, with an expanded sample size.

## 5 Conclusion

The study findings provide conclusive evidence regarding the prognostic significance of cytogenetic profiles determining the outcomes of AML patients undergoing allogeneic HSCT, independent of key factors such as age, sex, donor matching, time interval between diagnosis and transplantation, and disease status at the time of transplantation. Given the observed disparities between our study findings and established cytogenetic‐based risk stratifications, it is recommended to reevaluate existing guidelines and, if necessary, develop risk‐adapted approaches specifically tailored to Iranian AML patients undergoing HSCT. Furthermore, prospectively tracking survival outcomes for Iranian AML patients after allogeneic transplantation in clinical trials focused on specific cytogenetic or molecular abnormalities is advisable. Such trials would yield invaluable data that could be integrated into evidence‐based treatment guidelines for AML.

## Author Contributions

**Amir Abbas Rashidi:** conceptualization, methodology, writing – original draft, supervision, and project administration. **Amir Kasaeian:** conceptualization, methodology, investigation, project administration, and supervision. **Kamran Alimoghadam:** investigation, project administration, and supervision. **Maryam Noori:** writing – original draft, visualization, and validation. **Hediyeh Alemi:** writing – review and editing, validation, and visualization. **Ghazal Seghatoleslami:** writing – review and editing, validation, and visualization. **Oveis Salehi:** investigation, project administration, and supervision. **Ardeshir Ghavamzadeh:** investigation, project administration, and supervision. **Mohammad Vaezi:** investigation, project administration, and supervision. **Seyed Asadollah Mousavi:** data curation, formal analysis, and software. **Hossein Kamranzadeh:** writing – original draft, validation, and visualization. **Davood Babakhani:** investigation, project administration, and supervision. **Soroush Rad:** investigation, project administration, and supervision. **Maryam Barkhordar:** writing – original draft, validation, and resources. **Sahar Tavakoli Shiraji:** writing – review and editing, validation, and resources. **Marjan Yaghmaie:** conceptualization, methodology, writing – review and editing, supervision.

## Conflicts of Interest

The authors declare no conflicts of interest.

## Transparency Statement

The corresponding author, Marjan Yaghmaie, affirms that this manuscript is an honest, accurate, and transparent account of the study being reported; that no important aspects of the study have been omitted; and that any discrepancies from the study as planned (and, if relevant, registered) have been explained.

## Supporting information

Supplementary Information.

## Data Availability

The data that support the findings of this study are available from the corresponding author upon reasonable request.
