# Supplementary material for: Cytogenetic Abnormalities and Their Impact on Acute Myeloid Leukemia Outcomes Following Allogeneic Hematopoietic Stem Cell Transplantation: A Single‐Center Retrospective Study
Source: Health Sci Rep. 2025 Jun 20;8(6):e70914. doi: 10.1002/hsr2.70914 (PMC12180083; doi:10.1002/hsr2.70914)
Supplement: Supplementary file 1 — Supplementary Information. [file HSR2-8-e70914-s001.docx]

**Table S1.** Probability of overall survival for CBF-AML patients after excluding secondary abnormalities or complex karyotypes.

| Groups | Number | 1 year-OS  (95% CI) | 3 year-OS  (95% CI) | 5 year-OS  (95% CI) | P-value |
| --- | --- | --- | --- | --- | --- |
| **t (8;21)** | 6 | 80.0 (20.4-96.9) | 40.0 (5.2-75.3) | 40.0 (5.2-75.3) | 0.160 |
| **Inversion (16) & t (16;16)** | 7 | 85.7 (33.4-97.9) | 71.4 (25.8-92.0) | -- | 0.971 |

**Abbreviations:** OS: Overall survival; CI: Confidence interval; NA: Not available

**Table S2**. Univariate and multivariate analysis of overall survival for CBF-AML patients after excluding secondary abnormalities or complex karyotypes

| Groups | Number | Unadjusted HR  (95% CI) | P-value | Adjusted HR  (95% CI) | P-value |
| --- | --- | --- | --- | --- | --- |
| **t (8;21)** | 6 | 2.27 (0.69-7.44) | 0.172 | 2.70 (0.81-8.99) | 0.104 |
| **Inversion (16) & t (16;16)** | 7 | 1.02 (0.24-4.27) | 0.971 | 0.88 (0.19-3.94) | 0.876 |

**Abbreviations:** HR: Hazard ratio; CI: Confidence interval; NA: Not available

**Table S3.** Probability of leukemia free survival for CBF-AML patients after excluding secondary abnormalities or complex karyotypes

| Groups | Number | 1 year-LFS  (95% CI) | 3 year-LFS  (95% CI) | 5 year-LFS  (95% CI) | P-value |
| --- | --- | --- | --- | --- | --- |
| **t (8;21)** | 6 | 83.3 (27.3-97.5) | 41.7 (5.6-76.6) | 41.7 (5.6-76.6) | 0.226 |
| **Inversion (16) & t (16;16)** | 7 | 71.4 (25.8-92.0) | 71.4 (25.8-92.0) | -- | 0.981 |

**Abbreviations:** LFS: Leukemia free survival; CI: Confidence interval; NA: Not available

**Table S4**. Univariate and multivariate analysis of leukemia free survival for CBF-AML patients after excluding secondary abnormalities or complex karyotypes

| Groups | Number | Unadjusted HR  (95% CI) | P value | Adjusted HR  (95% CI) | P value |
| --- | --- | --- | --- | --- | --- |
| **t (8;21)** | 6 | 2.03 (0.62-6.62) | 0.236 | 2.36 (0.71-7.79) | 0.159 |
| **Inversion (16) & t (16;16)** | 7 | 0.98 (0.23-4.08) | 0.981 | 1.00 (0.24-4.16) | 0.998 |

**Abbreviations:** HR: Hazard ratio; CI: Confidence interval; NA: Not available
